# Supplementary material for: Digital economy development and global value chain network centralization
Source: PLoS One. 2025 Jul 17;20(7):e0328217. doi: 10.1371/journal.pone.0328217 (PMC12270168; doi:10.1371/journal.pone.0328217)
Supplement: S1 — (DOCX) [file pone.0328217.s001.docx]

**Appendix**

The 2016 version of the WIOD (World Input-Output Database) includes a total of 42 countries, which are as follows: Australia、Austria、Belgium、Bulgaria、Brazil、Canada、Switzerland、China、Cyprus、Czech Republic、Germany、Denmark、Spain、Estonia、Finland、France、United Kingdom、Greece、Croatia、Hungary、Indonesia、India、Ireland、Italy、Japan、Korea、Lithuania、Luxembourg、Latvia、Mexico、Malta、Netherlands、Norway、Poland、Portugal、Romania、Russia、Slovakia、Slovenia、Sweden、Turkey、United States.
