# Supplementary material for: Digital economy development and global value chain network centralization
Source: PLoS One. 2025 Jul 17;20(7):e0328217. doi: 10.1371/journal.pone.0328217 (PMC12270168; doi:10.1371/journal.pone.0328217)
Supplement: Attachment — Submitted filename: Response to Reviewers.docx [file pone.0328217.s003.docx]

**Response to Editor and Reviewers**

**Dear** **Editor and Reviewers:**

**First of all, please allow us to take this opportunity to express our heartfelt thanks to you for taking time out from your busy schedule to review this manuscript. You have provided us with constructive comments and suggestions, which are of great help for us to further improve this manuscript. We have carefully reviewed and revised the manuscript according to your valuable comments and suggestions. Here, we explain the revised work in detail below and provide the point-by-point responses to the editor’s and** **reviewers’ comments. Moreover, we have included continuous line numbers in the revised manuscript and have marked all variations in blue.**

**Response to Editor:**

**(1)** The introduction section should further clarify several questions: Why is the topic significant in the current economic landscape? What are the key research questions and problems? I would suggest the author enhance the theoretical argument. This will help readers who are not familiar with the topic to better understand the research objectives.

**Response:** Thank you for your specific and professional comments and suggestions. We have revised the introduction to emphasize the research significance and key questions of the article, as shown in blue below.

It is observed that enhancing the competitiveness of countries in global value chains requires relying on the support of high technology represented by the digital economy, this provides a rich practical significance and realistic background for the research of this paper.

Whereas the realization of GVC network centralization is a manifestation of countries' competitiveness in the GVC system; unfortunately, most studies currently focus on the drive of the digital economy for upgrading the GVC and have not yet deeply examined the impact of the digital economy on the centralization of the GVC network. Then, the key questions of this research are as follows: with the development of the global digital economy, what is its impact on the position of countries in the global value chain network? What kind of mechanism of action drives this impact? The answers to these questions are conducive to promoting the high-quality development of the manufacturing industry and realizing a higher level of integration into the global value chain network.

**(2)** The literature review should be enhanced by adding a separate section or more literature studies and providing relevant citations. It can be further enriched with up-to-date studies to motivate the readers in the background and currency of the subject of the research. Moreover, the authors should further clarify the gaps in the literature and how this research contributes to filling those gaps.

**Response:** Thank you for your specific and professional comments and suggestions. We have included the literature review as a separate section and added the corresponding literature. Our contribution to the existing research has also been added at the end of the literature review. The modifications are shown in blue below.

**Ⅱ. Literature Review**

The research related to this paper focuses on the following aspects: first, the changes in the division of production labor in today's GVC. The change in firms in terms of the division of production in GVC has been measured using the concept of the number of production stages[15]. Currently, the division of labor in GVC is facing many challenges, and COVID-19 has had a great impact on the global production supply chain [1,12,28], as it slowed the growth of the global economy and gradually reduced the incentive of countries to participate in the division of labor in GVC. The rise of artificial intelligence (AI) has had an impact on GVC, as the application of AI increased the efficiency of using intermediate goods to produce final goods [10], making the production process more automated and smarter, saving labor costs [5], and improving the division of labor of firms in GVC.

In contrast to the current literature, this paper investigates the impact of digital economy development on the centralization of GVC networks from theoretical and empirical aspects and makes innovations in the following three aspects.

First, this paper extends the heterogeneous firm trade model, introduces digital economy development factors in firms' production behavior and export decisions, and gauges firm behavior at the country level to construct a theoretical framework of the impact of digital economy development on the centralization of GVC networks.

Second, this paper uses country-industry level data to empirically study how digital economy development affects the centralization of the GVC network. Referring to Arnold et al. and Xiao et al., this paper uses the UIBE GVC Indicators database and the WIOD database to construct country-industry level digital economy development and GVC network centralization indicators [9,37]. Considering possible endogeneity problems, this paper refers to Nunn & Qian and uses the number of landline telephones in each country in 1984 as an instrumental variable for the least squares analysis [29].

Third, this paper summarizes two major channels through which the development of the digital economy affects the centralization of the GVC network, namely, the productivity channel and the resource allocation channel. Numerous studies have shown that digital economic development effectively reduces export fixed costs, while the digital economy has a significant substitution effect on labor and it increases labor productivity. With the increase in productivity, a country has a more competitive advantage in the GVC network. In addition, the development of the digital economy facilitates the free flow of production factors among national sectors, alleviating resource mismatch, enabling advantage gains for a country in global competition, and promoting such a country to the center of the GVC network.

**(3)** The methodology section is good; however, the authors should further clarify and justify their choice of methods used. In addition, the author should also explain the choice of the Fixed Effect (FE) model.

**Response:** Thank you for your specific and professional comments and suggestions. We state the reason for the choice of method, with modifications as shown in blue below.

**(i) Method selection and model setting**

Making a comprehensive assessment, this paper chooses the panel fixed-effects model (FE) to test the impact of digital economy development on the centralization of global value chain networks. There are three main reasons for choosing this method. First, from the data point of view, this paper mainly uses cross-country panel data from 2004-2014 for the study, which is in line with the conditions for the use of panel models. Second, compared with ordinary least squares (OLS) and the panel random effects model, the panel fixed effects model considers individual and time effects, making the parameter estimation more accurate. Third, this paper further uses the Hausman test to determine that it is more reasonable to use the panel fixed effects model. Therefore, this paper uses the panel fixed effects model as the main method of empirical research.

**(4)** Presentation of the summary of descriptive statistics in a tabulated form is also recommended, which is missing in the article.

**Response:** Thank you for your specific and professional comments and suggestions. We have added a descriptive statistics section to the revised draft. The modifications are shown in blue below.

**(2) Descriptive statistics**

Table 1 reports the descriptive statistics of the main variables of the paper.

**Table 1.** **Descriptive statistics**

| Variable | Observations | Mean | Std | Min | Max |
| --- | --- | --- | --- | --- | --- |
| centralization | 9625 | 6.8394 | 2.4994 | 0 | 12.8688 |
| Digit | 9625 | 0.2568 | 0.1891 | 0.0566 | 0.8807 |
| Labor | 9416 | 59.9074 | 6.2013 | 45.5233 | 74.2437 |
| Persons | 9625 | 4.5078 | 1.1999 | 1.2865 | 7.2145 |
| Scale | 9625 | 13.4145 | 3.9393 | 0 | 14.4201 |
| Fdi | 9625 | 2.5401 | 3.8481 | 0 | 14.2313 |
| GDP | 9625 | 0.0814 | 0.0041 | -0.0288 | 0.2345 |
| RTA | 9625 | 3.7367 | 0.8973 | 0.6931 | 4.4543 |

**(5)** The author should provide references and links for all the sources used in the research. For instance, the references for the UIBE database and WIOD database are missing.

**Response:** Thank you for your specific and professional comments and suggestions. Forgive us for not paying attention to this literature, which we have added to the revised manuscript and revised elsewhere accordingly. In addition, we also add more literature as support, as follows.

1. Timmer M P, Dietzenbacher E, Los B, Stehrer R, De Vries G J. An Illustrated User Guide to the World Input–Output Database: the Case of Global Automotive Production[J]. Review of International Economics, 2015, 23: 575–605.

2. Xiao H，Sun T，Meng B，Cheng L. Complex Network Analysis for Characterizing Global Value Chains in Equipment Manufacturing[J]. Plos One, 12 (1), e169549.

**(6)** The language of the paper needs careful editing and proofreading to improve readability.

**Response:** Thank you for your specific and professional comments and suggestions. We carefully proofread and optimize the language of writing. Here are the proofs：

**(7)** Please enhance the conclusion by further clarifying the limitations and future research directions.

**Response:** Thank you for your specific and professional comments and suggestions. We have added the limitations of the article and room for research directions in the conclusion section. The modifications are shown in blue below.

There are various limitations as well as the space that can be improved in the future. First, the object of this paper's research is GVC network centralization at the country-industry level, whereas in today's global world, enterprises are the main body participating in the competition of GVC, and the development of the digital economy effects on enterprises is the direction we need to study. Second, due to the availability of data, the research interval of this paper covers 2004-2014. However, with the continuous development of the digital economy, its long-term impact on the centralization of global value networks has to be further refined and analyzed after the database is updated.

**(8)** In your Data Availability statement, you have not specified where the minimal data set underlying the results described in your manuscript can be found.

**Response:** Thank you for your specific and professional comments and suggestions. We added the Data Availability statement:

Data Availability Statement

WIOD database: https://www.rug.nl/ggdc/valuechain/wiod/

UIBE GVC Indicators database: http://gvcdb.uibe.edu.cn/

all data are fully available without restriction

**Response to Reviewer #1:**

**(1)** In order to establish the significance and value of the study, it is necessary to provide a comprehensive rationale for the research, which emphasizes its relevance and unique contributions to the current scholarly discourse. This will strengthen the study's originality and scholarly impact. Following are useful suggested studies to get the benefit to update this part:

https://doi.org/10.1007/s11356022197186 ;

https://doi.org/10.3390/su14031054;

https://doi.org/10.1007/s1135602117438x;

https://doi.org/10.1016/j.resourpol.2022.102730 ;

https://doi.org/10.1108/FS-02-2021-0053;

https://doi.org/10.1002/ijfe.2073;

https://doi.org/10.1108/LHT-03-20210113;

https://doi.org/10.1007/s1135602221929w;

https://doi.org/10.3389/fpubh.2022.1009393;

https://doi.org/10.1002/sd.2529;

https://doi.org/10.3389/fenvs.2022.1068398;

https://doi.org/10.1016/j.techfore.2023.122413;

<https://doi.org/10.1007/s11356-022-20178-1>.

**Response:** Thank you for your specific and professional comments and suggestions. Forgive us for not paying attention to this literature, which we have added to the revised manuscript and revised elsewhere accordingly. In addition, we also add more literature as support, as follows:

1. Abbass K, Begum H, Alam ASAF, Awang AH, Abdelsalam MK, Egdair IMM, Wahid R. Fresh Insight through a Keynesian Theory Approach to Investigate the Economic Impact of the COVID-19 Pandemic in Pakistan[J]. Sustainability, 2022, 14(3):1054.

2. Acemoglu D, Restrepo P. The Race between Man and Machine：Implications of Technology for Growth，Factor Shares，and Employment［J］. American Economic Review，2018，108（6）：1488-1542.

3. Aghion P, Howitt P. The Economics of Growth［M］. Massuchusetts：The MIT Press，2008.

4. Begum H , Abbass K , Alam A S A F , Song H, Chowdhury MT，Ghani ABA. Impact of the COVID-19 pandemic on the environment and socioeconomic viability: a sustainable production chain alternative[J]. Foresight:The journal for future studies, strategic thinking and policy, 2022(3/4):24.

5. Fally，T. Production Staging：Measurement and Facts［R］. University of Colorado Boulder Working Paper，2012.

6. Naseer S, Khalid S, Parveen S, Abbass K, Song Hand Achim MV (2023) COVID-19 outbreak:Impact on global economy[J]. Frontiers in Public Health 2022, (10):1-13.

**(2)** The authors are requested to provide a thorough justification for the choice of the current methodology, including an explanation of the benefits and advantages it offers over alternative approaches. This will lend greater credibility and rigor to the study's research design.

**Response:** Thank you for your specific and professional comments and suggestions. We state the reason for the choice of method, with modifications as shown in blue below.

**(i) Method selection and model setting**

Making a comprehensive assessment, this paper chooses the panel fixed-effects model (FE) to test the impact of digital economy development on the centralization of global value chain networks. There are three main reasons for choosing this method. First, from the data point of view, this paper mainly uses cross-country panel data from 2004-2014 for the study, which is in line with the conditions for the use of panel models. Second, compared with ordinary least squares (OLS) and the panel random effects model, the panel fixed effects model considers individual and time effects, making the parameter estimation more accurate. Third, this paper further uses the Hausman test to determine that it is more reasonable to use the panel fixed effects model. Therefore, this paper uses the panel fixed effects model as the main method of empirical research.

**(3)** To highlight the novelty and contribution of the current study, it is necessary to outline the ways in which it diverges from the existing body of literature. Therefore, the concluding section of the literature review should include a clear differentiation of the study from prior research.

**Response:** Thank you for your specific and professional comments and suggestions. We have included the literature review as a separate section and added the corresponding literature. Our contribution to the existing research has also been added at the end of the literature review. The modifications are shown in blue below.

**Ⅱ. Literature Review**

The research related to this paper focuses on the following aspects: first, the changes in the division of production labor in today's GVC. The change in firms in terms of the division of production in GVC has been measured using the concept of the number of production stages[15]. Currently, the division of labor in GVC is facing many challenges, and COVID-19 has had a great impact on the global production supply chain [1,12,28], as it slowed the growth of the global economy and gradually reduced the incentive of countries to participate in the division of labor in GVC. The rise of artificial intelligence (AI) has had an impact on GVC, as the application of AI increased the efficiency of using intermediate goods to produce final goods [10], making the production process more automated and smarter, saving labor costs [5], and improving the division of labor of firms in GVC.

In contrast to the current literature, this paper investigates the impact of digital economy development on the centralization of GVC networks from theoretical and empirical aspects and makes innovations in the following three aspects.

First, this paper extends the heterogeneous firm trade model, introduces digital economy development factors in firms' production behavior and export decisions, and gauges firm behavior at the country level to construct a theoretical framework of the impact of digital economy development on the centralization of GVC networks.

Second, this paper uses country-industry level data to empirically study how digital economy development affects the centralization of the GVC network. Referring to Arnold et al. and Xiao et al., this paper uses the UIBE GVC Indicators database and the WIOD database to construct country-industry level digital economy development and GVC network centralization indicators [9,37]. Considering possible endogeneity problems, this paper refers to Nunn & Qian and uses the number of landline telephones in each country in 1984 as an instrumental variable for the least squares analysis [29].

Third, this paper summarizes two major channels through which the development of the digital economy affects the centralization of the GVC network, namely, the productivity channel and the resource allocation channel. Numerous studies have shown that digital economic development effectively reduces export fixed costs, while the digital economy has a significant substitution effect on labor and it increases labor productivity. With the increase in productivity, a country has a more competitive advantage in the GVC network. In addition, the development of the digital economy facilitates the free flow of production factors among national sectors, alleviating resource mismatch, enabling advantage gains for a country in global competition, and promoting such a country to the center of the GVC network.

**(4)** I would kindly request that the authors re-examine the policy implications derived from the study's findings, to ensure their accuracy and relevance to the study's objectives.

**Response:** Thank you for your specific and professional comments and suggestions. We have rewritten the policy implications section of this paper. The modifications are shown in blue below.

The findings of this paper have important policy implications. First, the digital economy is actively developing. The research in this paper finds that with the development of the digital economy, a country's position in the global value chain network continues to be centralized and moves up the value chain. Therefore, countries should continue to launch policies to support the development of the digital economy, improve the construction of digital infrastructure, and help enterprises realize digital transformation. Second, the digital economy should be relied on to transform the mode of development. The traditional economic growth mode driven by capital factors, natural resources and labor inputs is gradually being replaced by the growth mode driven by science, technology and innovation, and cutting-edge science and technology represented by the digital economy is becoming increasingly important in economic development. By promoting the rapid development of the digital economy, not only can it effectively reduce labor costs, but it can also alleviate a country's resource mismatch and build strong support for countries to participate in the competitive heights of the global value chain network.

**(5)** In order to ensure the relevance and timeliness of the study’s cited sources, it is recommended that the authors review and update the references section.

**Response:** Thank you for your specific and professional comments and suggestions. Forgive us for not paying attention to this literature, which we have added to the revised manuscript and revised elsewhere accordingly. In addition, we also add more literature as support, as follows:

1. Abbass K, Begum H, Alam ASAF, Awang AH, Abdelsalam MK, Egdair IMM, Wahid R. Fresh Insight through a Keynesian Theory Approach to Investigate the Economic Impact of the COVID-19 Pandemic in Pakistan[J]. Sustainability, 2022, 14(3):1054.

2. Acemoglu D, Restrepo P. The Race between Man and Machine：Implications of Technology for Growth，Factor Shares，and Employment［J］. American Economic Review，2018，108（6）：1488-1542.

3. Aghion P, Howitt P. The Economics of Growth［M］. Massuchusetts：The MIT Press，2008.

4. Begum H , Abbass K , Alam A S A F , Song H, Chowdhury MT，Ghani ABA. Impact of the COVID-19 pandemic on the environment and socioeconomic viability: a sustainable production chain alternative[J]. Foresight:The journal for future studies, strategic thinking and policy, 2022(3/4):24.

5. Fally，T. Production Staging：Measurement and Facts［R］. University of Colorado Boulder Working Paper，2012.

6. Naseer S, Khalid S, Parveen S, Abbass K, Song Hand Achim MV (2023) COVID-19 outbreak:Impact on global economy[J]. Frontiers in Public Health 2022, (10):1-13.

7. Timmer M P., Dietzenbacher E, Los B, Stehrer R, De Vries G J. An Illustrated User Guide to the World Input–Output Database: the Case of Global Automotive Production[J]. Review of International Economics, 2015, 23: 575–605.

**(6)** The study appears to employ an excessive amount of acronyms, which may impede comprehension for the wider readership of the journal. Thus, it is recommended that the authors avoid excessive usage of acronyms and instead use the full form of terms to ensure ease of understanding for readers."

**Response:** Thank you for your specific and professional comments and suggestions. We have modified the acronyms except for global value chains（GVC）, such as using the heterogeneous firm model instead of the MO model, and constant elasticity of substitution instead of CES.

**Response to Reviewer #2:**

**（1）**The paper includes the very detailed description of the applied quantitative model, however there are a few references in the text to underlying models (such as MO model, CES form) which are not explained for the readers, however at least a short footnote would be desirable.

**Response:** Thank you for your specific and professional comments and suggestions. We have modified the acronyms except for global value chains（GVC）, such as using the heterogeneous firm model instead of the MO model, and constant elasticity of substitution instead of CES.

**（2）**The quantitative analysis included 3 hypotheses, but the discussion of the results or the conclusions fails to come back to these hypotheses and discuss them in a "concentrated" way.

**Response:** Thank you for your specific and professional comments and suggestions. We have added sentences to the analysis of the regression results that are consistent with the hypotheses of this paper. It shows that the empirical results of this paper come back to the theoretical hypotheses. The modifications are shown in blue below.

Column (4) further controls industry-year fixed effects, and the coefficients of the core explanatory variables are still significantly positive at the 1% level, indicating that the development of the digital economy has a significant contribution to the degree of global value chain network centralization in a country. This verifies Hypothesis 1.

Thus, digital economy development promotes the centralization of a country in the GVC network by reducing the degree of resource mismatch. The above findings validate Hypotheses 2 and 3 in the theoretical part of the paper.

**（3）**In my opinion this would also strengthen the soundness of the results. The last part of the conclusions includes a couple of policy recommendations which are only very loosely related to the quantitative analysis presented in the paper. Please review/reconsider them and make them more related to the empirical evidences.

**Response:** Thank you for your specific and professional comments and suggestions. We have rewritten the policy implications section of this paper. The modifications are shown in blue below.

The findings of this paper have important policy implications. First, the digital economy is actively developing. The research in this paper finds that with the development of the digital economy, a country's position in the global value chain network continues to be centralized and moves up the value chain. Therefore, countries should continue to launch policies to support the development of the digital economy, improve the construction of digital infrastructure, and help enterprises realize digital transformation. Second, the digital economy should be relied on to transform the mode of development. The traditional economic growth mode driven by capital factors, natural resources and labor inputs is gradually being replaced by the growth mode driven by science, technology and innovation, and cutting-edge science and technology represented by the digital economy is becoming increasingly important in economic development. By promoting the rapid development of the digital economy, not only can it effectively reduce labor costs, but it can also alleviate a country's resource mismatch and build strong support for countries to participate in the competitive heights of the global value chain network.

**Finally, we sincerely thank you again for reviewing this manuscript and providing us with valuable comments and suggestions for revising and improving the manuscript. We look forward to hearing from you in due time regarding this submission and to respond to any further questions and comments you may have.**

**Yours sincerely**

**The Authors**

**August 2023**
