# Supplementary material for: Digital economy development and global value chain network centralization
Source: PLoS One. 2025 Jul 17;20(7):e0328217. doi: 10.1371/journal.pone.0328217 (PMC12270168; doi:10.1371/journal.pone.0328217)
Supplement: Attachment — Submitted filename: Response_to_Reviewers_auresp_2.docx [file pone.0328217.s004.docx]

**Response to Editor and Reviewers**

**Dear** **Editor and Reviewers:**

**First of all, please allow us to take this opportunity to express our heartfelt thanks to you for taking time out from your busy schedule to review this manuscript. You have provided us with constructive comments and suggestions, which are of great help for us to further improve this manuscript. We have carefully reviewed and revised the manuscript according to your valuable comments and suggestions. Here, we explain the revised work in detail below and provide the point-by-point responses to the editor’s and** **reviewers’ comments. Moreover, we have included continuous line numbers in the revised manuscript and have marked all variations in red.**

**Response to Editor:**

**(1)** The introduction section is clear, but it can be further developed. I would suggest the author to further enhance the theoretical argument.

**Response:** Thank you for your specific and professional comments and suggestions. We have added to the theoretical part of the introduction to strengthen the theoretical discussion:

In terms of theoretical research, this paper proves that the development of the digital economy promotes the centrality of the global value chain network by constructing a heterogeneous enterprise model, and that this promotion is mainly realized through the improvement of productivity and the reduction of resource allocation efficiency. With the development of the digital economy, it creates advantages for enterprises to participate in global value chain competition, and therefore promotes the country's production and cooperation in the global value chain network, and promotes its gradual development towards the center of the global value chain network.

**(2)** Please enhance the literature further and provide more relevant references for some contemporary literature works.

**Response:** Thank you for your specific and professional comments and suggestions. We have added the references accordingly in the References section, and the specific changes are described in the References section.

**(3)**The authors should revise the language and formatting of the article. Please follow the guidelines of PLOS ONE for all the headings and sub-headings as well as uniformity of the text and tables.

**Response:** Thank you for your specific and professional comments and suggestions. We have prepared all headings and sub-headings in accordance with PLOS ONE guidelines and harmonized the text and tables. The changes are shown in the revised version.

**Response to Reviewer :**

**(1**)The introduction section needs to be strengthened, and the most important thing is to highlight the research background, theoretical framework, and existing research deficiencies in the introduction section.

**Response:** Thank you for your specific and professional comments and suggestions. We make additions to the introduction section, mainly adding the theoretical framework and existing research deficiencies. The specific modifications are as follows:

In terms of theoretical research, this paper proves that the development of the digital economy promotes the centrality of the global value chain network by constructing a heterogeneous enterprise model, and that this promotion is mainly realized through the improvement of productivity and the reduction of resource allocation efficiency. With the development of the digital economy, it creates advantages for enterprises to participate in global value chain competition, and therefore promotes the country's production and cooperation in the global value chain network, and promotes its gradual development towards the center of the global value chain network.

This paper explores the impact of the development of the digital economy on the centralization of GVC networks mainly through theory and empirical evidence, and we find that, first, the digital economy significantly enhances the status of countries in GVC networks. Second, this enhancing effect varies with the degree of economic development and institutional environment of each country. Third, the development of the digital economy achieves the centralization of GVC networks through productivity enhancement and resource mismatch mitigation. Fourth, the digital economy effectively enhances a country's breadth and depth in global value chains. In addition, there is room to expand the research in this paper, such as considering the impact of GVC network centrality from the firm level.

**(2)** The conclusion section of the study still needs to be strengthened. In addition to summarizing this article, the conclusion of the study also needs to be discussed in relation to existing relevant research. And the differences between your research findings and existing related research.

**Response:** Thank you for your specific and professional comments and suggestions. We have modified the findings of the study to emphasize the linkage of the findings to existing research, as described below:

In contrast to existing research, which focuses more on the impact of the digital economy on the upgrading of GVCs and ignores the impact on the centrality of GVC networks, the findings of this paper are an important addition in this regard.

**(3)** I think the policy recommendations in this manuscript are not sufficient, It is recommended to refer to or cite this paper: <https://doi.org/10.1016/j.resourpol.2023.104174>

**Response:** Thank you for your specific and professional comments and suggestions. We have cited the above literature in our policy recommendations. The specific changes are set out below:

The traditional mode of economic growth driven by capital factors, natural resources and labor inputs is gradually being replaced by the mode of growth driven by science and technology and innovation. Frontier science and technology, represented by the digital economy, has become increasingly important in economic development, such as the use of digital finance to continuously optimize the efficiency of resource allocation[38].

38. Huang A Z., Bi Q X., Dai L T., et al. Investigating the impact of financial development on the resource curse form its dual effect[J]. Resources Policy, 2023,86(10):1403-1448.

**(4)** It is suggested to further emphasize the significance of GVC network centralization in the introduction.

**Response:** Thank you for your specific and professional comments and suggestions. We have emphasized the significance of GVC networks centralization in the introduction. Specific concrete changes are described below:

The global value chain network not only reflects the overall world trade pattern and trade connections but also demonstrates the importance of countries in global trade and whether they are in pivotal positions. If a country is positioned more centrally in the trade network, it signifies a stronger ability to control and access resources and information.

**(5)** It is suggested to focus on the impact of ICT, network infrastructure, informatization and other economic variables on GVC in the literature review, the existing references pay less attention to the core issues of this paper

**Response:** Thank you for your specific and professional comments and suggestions. We incorporated the impacts of information and communication technology, network infrastructure, informatization, and other economic variables on the global value chain in our literature review. The specific modifications are as follows:

Additionally, research suggests that information and communication technology (ICT) plays a significant role in driving global value chain (GVC) activities[19]. Studies from both macro and micro perspectives have found that the development of the internet notably promotes GVC trade[20]. This promotion is mainly achieved by enhancing supply-demand matching and fostering communication and collaboration, injecting new vitality into the global value chain[21]. However, there are also opinions suggesting that ICT may have adverse effects on the upgrading of GVCs. Under the global value chain division system driven by labor arbitrage, the substitution effect of automation on labor leads to the contraction of GVC trade[22].

19. Freund C., Weinhold D. The Internet and International Trade in Services[J]. American Economic Review, 2002, 92(2):236-240.

20. Xing Z W. The Impacts of Information and Communications Technology (ICT) and E-commerce on Bilateral Trade Flows[J]. International Economics and Economic Policy, 2018, 15(3):565-586.

21. Antràs P. Conceptual Aspects of Global Value Chains[J]. World Bank Economic Review, 2020, 34(3):551-574.

22. Artuc E., Bastos P., Rijkers B. Robots, Tasks and Trade[R]. Policy Research Working Paper, 2018, No.8674.

**Finally, we sincerely thank you again for reviewing this manuscript and providing us with valuable comments and suggestions for revising and improving the manuscript. We look forward to hearing from you in due time regarding this submission and to respond to any further questions and comments you may have.**

**Yours sincerely**

**The Authors**

**Apr 2024**
