# Supplementary material for: Digital economy development and global value chain network centralization
Source: PLoS One. 2025 Jul 17;20(7):e0328217. doi: 10.1371/journal.pone.0328217 (PMC12270168; doi:10.1371/journal.pone.0328217)
Supplement: Attachment — Submitted filename: Response_to_Reviewers_auresp_3.docx [file pone.0328217.s005.docx]

**Response to Editor and Reviewers**

**Dear** **Editor and Reviewers:**

**First of all, please allow us to take this opportunity to express our heartfelt thanks to you for taking time out from your busy schedule to review this manuscript. You have provided us with constructive comments and suggestions, which are of great help for us to further improve this manuscript. We have carefully reviewed and revised the manuscript according to your valuable comments and suggestions. Here, we explain the revised work in detail below and provide the point-by-point responses to the editor’s and** **reviewers’ comments. Moreover, we have included continuous line numbers in the revised manuscript and have marked all variations in red.**

**Response to Editor:**

**(1)** Please enhance the literature further and provide more relevant references for some contemporary literary works.

**Response:** Thank you for your specific and professional comments and suggestions. The authors followed the reviewer's comments and added appropriate literature to enhance the scholarly nature of the paper, which included:

Caliendo, L., & Parro, F. (2015). Estimates of the Trade and Welfare Effects of NAFTA. The Review of Economic Studies, 82(1), 1-44.
 Melitz, M. J., & Ottaviano, G. I. (2008). Market size, trade, and productivity. The Review of Economic Studies, 75(1), 295-316.

**(2)** There are minor grammatical errors. The authors should revise the language and formatting of the article.

**Response:** Thank you for your specific and professional comments and suggestions. The corresponding grammar and expression have been modified in this paper, specifically as follows:

Most of the studies mentioned above have focused on the impact of the digital economy on the "binary margin" of firms' exports,（page 11, line 101）

but it also has a substitution effect on the traditional labor force in terms of creating new jobs（page 11, line 96）

α and η represent the elasticity of substitution between homogeneous and heterogeneous products（page 13, line 184.）

$q_{i}$ represents a heterogeneous product（page 13, line 183）

$\Omega$ is a set of indexes（page 13, line 185）

$\gamma$ represents the degree of product differentiation between the varieties （page 13, line 183）

**Response to Reviewer :**

**(1)** **Regarding the Theory:**

The authors depart from the basic 2-product CES production function by adding another production input. However, what is the contribution of this addition? What makes the digital economy so special and unique? Is the digital economy an actual factor of production, or is it more of an intermediate product? A simpler 2-good model, where the digital economy acts as an intermediate good used to produce a final product for exports, might have been more convincing. However, similar models (with intermediate goods) have been explored in other contexts (e.g., Caliendo and Parro, 2015), leaving the question of what makes the digital economy unique unanswered.

**Response:** Thank you for your specific and professional comments and suggestions. In the original model, this paper regards the digital economy as a production factor input into the production of firms. As the reviewer said, it is more appropriate to put the digital economy into production as an intermediate. Therefore, this paper refers to the practice of Caliendo and Parro (2015) and puts the digital economy into production as an intermediate. But the authors also want to emphasize that whether the digital economy is put into production as a factor of production or an intermediate, the conclusion that the development of the digital economy affects the centralization of the global value chain network by lowering the price of digital factors will not change. Therefore, the core conclusion of this paper will not change. Specific modifications to the model are as follows:

According to the practice of Caliendo and Parro, the firm production function is set as follows:

$y\left( \varphi\right)=z(\varphi)\left[ l(\varphi) \right]^{\gamma^{l}}\left[ s(\varphi) \right]^{\gamma^{s}}\prod_{k=1}^{n} \left[ m(\varphi) \right]^{\gamma^{k}}$	（5）

Equations (5) represents the final product produced by the enterprise is composed of a series of intermediate products, $z(\varphi)$ represents the productivity of the firm. $l(\varphi)$ represents labor input. $s(\varphi)$ represents digital technology. $m(\varphi)$ represents the input of a series of other intermediate goods. $\gamma^{l}$, $\gamma^{s}$, and $\gamma^{k}$ represent the input shares of labor, digital technology, and other intermediate goods, respectively. According to the principle of firm cost minimization, the firm's marginal cost is:

$c=\Upsilon w^{\gamma^{l}}p_{s}^{\gamma^{s}}\prod_{k=1}^{n} {(p_{m})}^{\gamma^{k}}$	（6）

Where $\Upsilon=\left( \gamma^{l} \right)^{-\gamma^{l}}\left( \gamma^{s} \right)^{-\gamma^{s}}\prod_{k=1}^{n} {(\gamma^{k})}^{\gamma^{-k}}$. $w$, $p_{s}$ and $p_{m}$ represent wage price, digital technology price and other intermediate goods price respectively.

**(2)** **Regarding the Empirical Analysis:**

At the macroeconomic level, establishing causality is challenging, if not impossible, at least in its contemporary sense. This limitation is not the authors' fault, but it should be addressed in the study. While it is valid to empirically test the relationships derived from a theoretical model, the text should acknowledge this limitation. The authors should neither state nor imply causality. For instance, the second sentence of the abstract reads: “this paper […] empirically examines the impact of digital economy development on the centralization of the GVC network and the internal mechanism.” There are several other instances where causality is either stated or implied.

**Response:** Thank you for your specific and professional comments and suggestions. This paper should not state or imply causality between macro variables in the abstract. Therefore, the paper describes the relationship between the two variables in the abstract as:

empirically examines the effects of digital economy development on the centralization of the GVC network and the internal mechanisms.

**(3) Minor Comments:**

There are several typos. For example, on page 11, line 101, “mentined” should be “mentioned.”

There are also several punctuation errors, such as a missing space after “jobs” on page 11, line 96, and missing spaces between $alpha$ and $\eta$ on page 13, line 184.
 The mathematical notation contains inaccuracies. For instance, on page 13, line 183, $q_i$ is not a set but a heterogeneous good. Additionally, the way the integral is written implies that $\Omega$ is a set of indexes $i$, not a set of goods.
 Quoting Melitz and Ottaviano (2008), “the parameter $\gamma$ indexes the degree of product differentiation between the varieties,” which is neither defined as an elasticity nor “happens to be equal” to an elasticity. Since the demands are linear (due to quadratic utility), the elasticities are definitely not constant.

**Response:** Thank you for your specific and professional comments and suggestions. The corresponding grammar and expression have been modified in this paper, specifically as follows:

Most of the studies mentioned above have focused on the impact of the digital economy on the "binary margin" of firms' exports,（page 11, line 101）

but it also has a substitution effect on the traditional labor force in terms of creating new jobs（page 11, line 96）

α and η represent the elasticity of substitution between homogeneous and heterogeneous products（page 13, line 184.）

$q_{i}$ represents a heterogeneous product（page 13, line 183）

$\Omega$ is a set of indexes（page 13, line 185）

$\gamma$ represents the degree of product differentiation between the varieties （page 13, line 183）

**Finally, we sincerely thank you again for reviewing this manuscript and providing us with valuable comments and suggestions for revising and improving the manuscript. We look forward to hearing from you in due time regarding this submission and to respond to any further questions and comments you may have.**

**Yours sincerely**

**The Authors**

**Sep 2024**
