# Supplementary material for: Digital economy development and global value chain network centralization
Source: PLoS One. 2025 Jul 17;20(7):e0328217. doi: 10.1371/journal.pone.0328217 (PMC12270168; doi:10.1371/journal.pone.0328217)
Supplement: Attachment — Submitted filename: Response_to_Reviewers_auresp_4.docx [file pone.0328217.s006.docx]

**Response to Editor and Reviewers**

**Dear Editor and Reviewers:**

**First of all, please allow us to take this opportunity to express our heartfelt thanks to you for taking time out from your busy schedule to review this manuscript. You have provided us with constructive comments and suggestions, which are of great help for us to further improve this manuscript. We have carefully reviewed and revised the manuscript according to your valuable comments and suggestions. Here, we explain the revised work in detail below and provide the point-by-point responses to the editor’s and reviewers’ comments. Moreover, we have included continuous line numbers in the revised manuscript and have marked all variations in red.**

**In response to the comments raised by the reviewers, the authors have made detailed revisions. Firstly, we would like to briefly report to the review experts the main content of this revision: The revision primarily focuses on the theoretical model and empirical research, particularly the discussion on causality. In the revision of the theoretical model, data elements in the digital economy are mainly introduced as production factors into enterprises' production decisions, influencing their operational performance. This emphasizes the unique position and role of the digital economy within the model. In the revision of the empirical research, we have conducted supplementary discussions on the causality between the development of the digital economy and the centralization of global value chain networks. This includes adding correlation coefficient tests and employing the instrumental variable approach to address potential endogeneity issues. However, the authors also acknowledge that their discussion on causality is only based on our best efforts to analyze, and there is certainly room for improvement. Therefore, further research on the relationship between the development of the digital economy and the centralization of global value chain networks will be a direction for the authors' future endeavors. Thank you for the understanding and inclusiveness.**

**Response to Reviewer :**

**（1）Regarding the Theory:**

**Reviewer’s comment:** I emphasized that an original research paper in macroeconomic theory should propose a model that explicitly demonstrates how the digital economy differs from other inputs or intermediate goods, thereby adding new insights to the literature. Instead, the authors appear content with adapting existing models by simply renaming variables, resulting in a theoretical contribution that is minimal or even absent.

**Authors’ response:** We appreciate the reviewer's comments and have revised the theoretical section accordingly. In particular, we have emphasized the distinction between the digital economy and other economic factors, as well as their specific impacts on overall corporate production decisions. Therefore, in the revised theoretical model, we have retained the basic framework of Melitz & Ottaviano (2008) and extended the endogenous growth model of De Ridder (2021) to construct a theoretical framework for corporate export behavior incorporating data as a factor of production.

The most significant modification in our model is the inclusion of data as a production factor in corporate production decisions, which further influences corporate operating performance. This underscores the unique role and importance of the digital economy within the model.

Why can data within the digital economy serve as a production factor influencing corporate production decisions? The reason is that the digital economy relies on data as a key production factor, with digital technology as its core driving force and modern information networks as its important carrier. Through the deep integration of digital technology with the real economy, it continuously enhances the digitization, networking, and intelligence levels of the economy and society, accelerating the reconstruction of economic development and governance models. Data is a crucial production factor in the digital economy era, often compared to coal and oil in the digital age. Firstly, as a new input, data can drive economic growth on its own. Unlike traditional production factors such as capital and labor, data has characteristics such as extremely low search costs, zero replication costs, extremely low transmission costs, and non-excludability (Goldfarb & Tucker, 2019). Data not only has nearly infinite supply but also has a wide range of applications, thus being able to break through the growth constraints of traditional production factors. Mainstream economic growth models have begun to introduce variables related to the digital economy, such as data capital and robots (Acemoglu & Restrepo, 2018). Secondly, digital technology enhances capital and labor factors, increasing their marginal output. From a capital perspective, the diffusion of digital technology can reshape a firm's production and management methods, enhancing the efficiency of capital accumulation. Therefore, in the current digital economy era, data as a key factor is a fundamental characteristic of the digital economy, continuously influencing corporate production decisions.

In the producer model, we assume that firms require labor input ($l_{i}$) and data capital ($f_{i}$) to produce goods. Since big data enables firms to make more scientific production decisions, we assume that data capital investment can reduce firms' marginal costs ($s_{i}$). That is, $c_{i}=s_{i}\omega$, where $s_{i}\in\left[ 0,1 \right]$, $\omega$ stands for wage level Hence, the production function of the firm can be expressed as:

$y_{i}=\frac{l_{i}}{s_{i}}$	（5）

The combination of data capital and labor has two characteristics in terms of its impact on output: 1. There are differences in the efficiency of different firms in developing and utilizing data capital, $\varphi_{i}$represented by firm-specific cost parameters. Due to the economies of scale and scope of data factors, cost parameters $\varphi_{i}$ significantly decrease with increased data capital investment $f_{i}$. 2. Firms invest in data capital before observing their competitors' marginal costs and pricing. When firms set prices and make production decisions, data assets have already become sunk costs.

The functional relationship between a firm's data capital $f_{i}$ and the decrease in marginal cost $s_{i}$ is given by $f\left( s_{i}，\varphi_{i} \right)$. The function $f_{i}$is twice differentiable, strictly decreasing when $s_{i}\in[0,1]$, and increasing when $\varphi_{i}>0$. It is assumed that $f\left( s_{i}，\varphi_{i} \right)$ is convex and decreasing in $s_{i}$, and satisfies $f\left( 1，\varphi_{i} \right)=0$，$\lim_{s_{i}\to0} f\left( s_{i}，\varphi_{i} \right)=\infty$. For the purpose of analysis, this paper selects an exponential function form for the data capital function that meets these characteristics:

$f\left( s_{i}，\varphi_{i} \right)=\varphi_{i}\left( \left[ \frac{1}{s_{i}} \right]^{\psi}-1 \right)$	（6）

Where $\psi>0$ represents the curvature parameter.

Subsequently, the decision-making process for the firm unfolds as follows: First, the firm decides on its data capital expenditure $f_{i}$, that is, whether to produce at a marginal cost lower than the benchmark $\omega$; then, the firm select $s_{i}$and incurs the corresponding data capital expenditure $f_{i}$; finally, the firm observes its competitors' marginal costs and sets its price $p_{i}$. The analysis will proceed using backward induction.

Decision on Data Capital Investment: Combining Equation (4), the$s_{i}$ that minimizes marginal cost at the optimal output $y_{i}^{*}$ is:

$s_{i}^{*}=min\left[ \left( \left[ y_{i}^{*} \right]^{-1}\psi\varphi_{i} \right)^{\frac{1}{\psi+1}}，1 \right]$	（7）

The modeling approach for producer behavior can be summarized as follows: With the development of the digital economy, data elements $f_{i}$are invested as important production factors in enterprise production, and as the digital economy becomes more developed, the marginal cost $s_{i}$ of enterprises decreases.

**(2) Regarding the Empirical Analysis:**

**Reviewer’s comment:** "The authors are empirically testing relationships derived from a theoretical model. There is nothing wrong with this approach, but the text should acknowledge its limitations: the authors should neither claim nor imply causality. For example, the second sentence of the abstract reads…" Although the abstract has been revised, the remainder of the paper continues to imply causality. For instance, section 5.2 is devoted to discussing endogeneity.

**Authors’ response:** We appreciate the reviewer's constructive comments and respond to the reviewer's concerns as follows: As the reviewer mentioned, the reviewer's questions primarily focus on the causality between the two variables selected in this paper, namely, whether the development of the digital economy leads to centralization in global value chain networks.

We clarify our writing approach: Firstly, in the theoretical analysis, we established a heterogeneous firm model and constructed a theoretical framework for corporate export behavior incorporating data as a factor of production. In the theoretical section, we found that the development of the digital economy enhances a country's central position in global value chain networks. Subsequently, this paper empirically tests the conclusions of the theoretical analysis. In the empirical tests, we first supplemented the correlation coefficient test, with the results shown in Table 2, revealing a positive correlation between the development of the digital economy and centralization in global value chain networks. Then, we proceed to assess the causality between the development of the digital economy and centralization in global value chain networks.

**Table 2. Correlation coefficient test.**

|  | centralization | Digit | Labor | Persons | Scale | Fdi | GDP | RTA |
| --- | --- | --- | --- | --- | --- | --- | --- | --- |
| centralization | 1.000 |  |  |  |  |  |  |  |
| Digit | 0.056^***^ | 1.000 |  |  |  |  |  |  |
| Labor | 0.162^***^ | 0.002 | 1.000 |  |  |  |  |  |
| Persons | -0.072^***^ | -0.001 | -0.364^***^ | 1.000 |  |  |  |  |
| Scale | 0.100^***^ | 0.000 | 0.077^***^ | 0.058^***^ | 1.000 |  |  |  |
| Fdi | -0.025^**^ | 0.066^***^ | 0.127^***^ | 0.000 | -0.325^***^ | 1.000 |  |  |
| GDP | 0.432^***^ | -0.002 | 0.343^***^ | 0.163^***^ | 0.207^***^ | 0.024^**^ | 1.000 |  |
| RTA | 0.099^***^ | 0.013 | 0.387^***^ | 0.183^***^ | 0.391^***^ | 0.303^***^ | 0.276 | 1.000 |

As the reviewer mentioned, judging causality is one of the difficulties in current economic research, and the biggest challenge in judging the causality between two variables lies in the issue of endogeneity. Endogeneity mainly manifests as omitted variables and reverse causality. In the baseline regression tests, we have added control variables as much as possible and found that whether control variables are included or not, the coefficients and significance of the core explanatory variables do not change significantly, indicating that the issue of omitted variables in this paper is relatively minor. Therefore, the main challenge in determining the causality between the development of the digital economy and centralization in global value chain networks lies in addressing reverse causality. The primary method in current economic research to address reverse causality is the use of instrumental variables. In our original research, we referred to the study by Nunn & Qian and used the number of telephone lines worldwide in 1984 as an instrumental variable for the development of the digital economy. The conclusions in Table 4 support the baseline regression results.

However, to make our research results more reliable and the causality between the development of the digital economy and centralization in global value chain networks more credible, in the revised manuscript, we have selected another instrumental variable to address potential endogeneity issues. Reference to Beverelli et al. (2017), we constructed a weighted value of the digital economy development level of other countries relative to the home country and the average digital economy development level of countries at different income levels, multiplied by the digitalization rate of various industries in each country, serving as an instrumental variable for the digital economy at the "country-industry" level. The rationale for this "country-industry" level digital economy instrumental variable is as follows: In terms of relevance, countries with similar economic development levels tend to have similar levels of digital economy development, satisfying the relevance requirement. In terms of exogeneity, the average level of digital economy development across different income levels reflects the digital economy development within that income bracket but does not contain country-specific development characteristics. Therefore, this instrumental variable meets the requirements. Table 5 shows the regression results using the "country-industry" level instrumental variable, indicating that after addressing potential endogeneity issues, the conclusion that the development of the digital economy enhances centralization in global value chain networks still holds.

**Table 5. Regression results of instrumental variables at the "country-industry" level.**

|  | **（1）** | **（2）** |
| --- | --- | --- |
|  | **2SLS** | **2SLS** |
| Digit | 0.0987^***^(0.0131) | 0.0881^***^(0.0134) |
| Control variables | NO | YES |
| Country fixed effects | YES | YES |
| Time fixed effects | YES | YES |
| Industry - year fixed effects | YES | YES |
| Kleibergen-Paap rk LM Statistics | 2.8e+06^***^[0.000] | 2.7e+06^***^[0.000] |
| Cragg-Donald Wald F Statistics | 3.2e+06{16.38} | 3.4e+06{16.38} |
| Kleibergen-Paap rk Wald FStatistics | 2.7e+06{16.38} | 2.8e+06{16.38} |
| N | 9625 | 8818 |

**Through the above series of tests, we have basically concluded that the development of the digital economy enhances centralization in global value chain networks. However, as the reviewer pointed out, verifying the causality between two variables is a difficult task in economic research, and we have also made every effort to study the impact of the development of the digital economy on centralization in global value chain networks. We acknowledge that there is room for improvement in our current research, which is also a direction we will strive towards in the future. We thank the reviewer for your understanding and consideration.**

**Finally, we sincerely thank you again for reviewing this manuscript and providing us with valuable comments and suggestions for revising and improving the manuscript. We look forward to hearing from you in due time regarding this submission and to respond to any further questions and comments you may have.**

**Yours sincerely**

**The Authors**

**Dec 2024**
