# Supplementary material for: Digital economy development and global value chain network centralization
Source: PLoS One. 2025 Jul 17;20(7):e0328217. doi: 10.1371/journal.pone.0328217 (PMC12270168; doi:10.1371/journal.pone.0328217)
Supplement: Attachment — Submitted filename: Response_to_Reviewers_auresp_5.docx [file pone.0328217.s007.docx]

**Response to Editor and Reviewers**

**Dear** **Editor and Reviewers:**

**First of all, please allow us to take this opportunity to express our heartfelt thanks to you for taking time out from your busy schedule to review this manuscript. You have provided us with constructive comments and suggestions, which are of great help for us to further improve this manuscript. We have carefully reviewed and revised the manuscript according to your valuable comments and suggestions. Here, we explain the revised work in detail below and provide the point-by-point responses to the editor’s and** **reviewers’ comments. Moreover, we have included continuous line numbers in the revised manuscript and have marked all variations in red.**

**Response to Editor:**

**(1)** Please enhance the title of the study. In the abstract, replace "high-quality opening up" with standard terms (e.g., "economic liberalization").

**Response:** Thank you for your specific and professional comments and suggestions. We have made corresponding revisions to the wording in the abstract, replacing "high-quality opening up" with "economic liberalization".

**(2)** The literature review needs to be enhanced, and more up-to-date literature studies should be added.

**Response:** Thank you for your specific and professional comments and suggestions. We have incorporated the latest literature in the literature review section of the revised manuscript. The newly added references encompass:

44. Fan Z , Zhou Y , Anwar S .Centralization of trade agreements network and global value chain participation[J].Quarterly Review of Economics and Finance, 2024, 94:11-24.

45. Li H , Yang Z .Does digital economy development affect urban environment quality: Evidence from 285 cities in China[J].PLoS ONE, 2024, 19(2):26.

**(3)** In the result section, please explain if the Hausman test was used to determine the model specification and add the chi-squared value in the table.

**Response:** Thank you for your specific and professional comments and suggestions. We have supplemented the results of the Hausman test in the revised manuscript. The results show that the P-value of the Hausman test is less than 0.01, leading to the rejection of the null hypothesis of random effects. Therefore, a fixed effects regression model should be selected for the analysis.

**Table 3. Baseline regression results of the development of the digital economy on the centralization of the value chain network.**

|  | **（1）** | **（2）** | **（3）** | **（4）** |
| --- | --- | --- | --- | --- |
|  | **FE** | **FE** | **FE** | **FE** |
| Digit | 0.0837^***^(0.0294) | 0.1990^***^(0.0342) | 0.0857^***^(0.0310) | 0.0876^***^(0.0325) |
| Labor |  | 0.0018(0.0073) | -0.0016(0.0069) | -0.0015(0.0067) |
| Persons |  | -2.5502^***^(0.5741) | -2.1943^***^(0.5575) | -2.2008^***^(0.5537) |
| Scale |  | 1.1061^***^(0.3982) | 1.2752^***^(0.3740) | 1.2734^***^(0.3712) |
| Fdi |  | -0.0017(0.0035) | -0.0031(0.0032) | -0.0031(0.0032) |
| GDP |  | 0.0814^**^(0.0330) | 0.1022^***^(0.0345) | 0.1024^***^(0.0348) |
| RTA |  | 0.0448(0.0328) | 0.0687^**^(0.0321) | 0.0683^**^(0.0338) |
| Constant term | 6.688^***^(0.0682) | 15.142^***^(2.6211) | 13.293^***^(2.5762) | 13.324^***^(2.5692) |
| Country fixed effects | YES | YES | YES | YES |
| Industry fixed effects | YES | NO | YES | NO |
| Hausman Test | 13.54（p=0.0003） | 14.89（p=0.0001） | 18.99（p=0.000） | 12.06（p=0.0006） |
| N | 9625 | 8818 | 8818 | 8818 |
| R^2^ | 0.632 | 0.380 | 0.630 | 0.632 |

**(4)** What does UIBE stand for? Please add it for the clarity of the readers.

**Response:** Thank you for your specific and professional comments and suggestions. We have clarified the meaning of "UIBE" (University of International Business and Economics) at its first occurrence in the revised manuscript. Additionally, we have provided a detailed description of the "UIBE GVC Indicators database" in the "Data description" section under 4.3.1.

(2) UIBE GVC Indicators database

The UIBE GVC Indicators Database is an open-access database constructed and maintained by the Global Value Chain Institute at the University of International Business and Economics (UIBE). This database is designed to provide useful indicators and data support for international trade research and global value chain (GVC) analysis.The UIBE GVC Indicators database is based on the input‒output data of countries and regions in the world and utilizes the structural decomposition method of input‒output to calculate the indicators of value-added trade and GVC participation, which are used to calculate the core explanatory variables of this paper[2].

**(5)** Please further justify the use of the time frame 2004-2014. Why not till 2024 or 2023?

**Response:** Thank you for your specific and professional comments and suggestions. The reason the sample period in this study only extends up to 2014 is due to the limitations of the database used. The calculation of the core variables in this paper relies on the WIOD (World Input-Output Database), which, as of now, has only been updated up to the year 2014. Furthermore, the data from 2004 to 2014 used in this study are sufficient to essentially demonstrate the conclusions of our research questions. Of course, once the WIOD database is updated in the future, we will also update the sample data for subsequent research. We hope the editor can understand this limitation.

**(6)** Further explain the selection and number of countries used and provide a list of the selected countries in the appendix.

**Response:** Thank you for your specific and professional comments and suggestions. In the appendix section of the revised manuscript, we have provided a detailed explanation of the data on the number of countries used in our study and listed the specific names of these countries.

Appendix

The 2016 version of the WIOD (World Input-Output Database) includes a total of 42 countries, which are as follows: Australia、Austria、Belgium、Bulgaria、Brazil、Canada、Switzerland、China、Cyprus、Czech Republic、Germany、Denmark、Spain、Estonia、Finland、France、United Kingdom、Greece、Croatia、Hungary、Indonesia、India、Ireland、Italy、Japan、Korea、Lithuania、Luxembourg、Latvia、Mexico、Malta、Netherlands、Norway、Poland、Portugal、Romania、Russia、Slovakia、Slovenia、Sweden、Turkey、United States.

**(7)** If possible, add a mechanism diagram or the research theoretical framework.

**Response:** Thank you for your specific and professional comments and suggestions. We have supplemented and drawn the theoretical framework diagram in the revised manuscript.

Figure 1 illustrates the mechanism through which the development of the digital economy influences the centralization of global value chain networks. Specifically, the integration of data elements into firms' production activities enhances the centralization of global value chain networks by improving productivity and alleviating resource misallocation through these two channels.

Fig. 1 A Diagram Illustrating the Mechanism by Which the Development of the Digital Economy Influences the Centralization of Global Value Chain Network

**(8)** Replace or rephrase vague phrases and overstated claims throughout the manuscript.

**Response:** Thank you for your specific and professional comments and suggestions. We have revised the expressions in this paper in the revised manuscript, removing vague phrases and overstated claims.

**Response to Reviewer6 :**

**(1)** **Clarify Theoretical Contributions**

The paper extends existing models (Melitz & Ottaviano, De Ridder) but could more explicitly highlight how its contributions differ from prior work.

Clearly articulate how introducing data as a production factor changes the implications for global value chain (GVC) centralization.

**Response:** Thank you for your specific and professional comments and suggestions. Firstly, regarding the theoretical contributions of this paper, our theoretical framework is derived from the Melitz & Ottaviano model, yet it distinctly differs from it. The most significant contribution lies in the expansion of the Producer behavior aspect. The Melitz & Ottaviano model primarily assumes that firms produce using only labor as a factor of production.. In contrast, this paper argues that in addition to the traditional factor of labor, data capital is also a crucial factor of production. This contribution not only extends the Producer behavior in the Melitz & Ottaviano model but also lays a theoretical foundation for our analysis of how the development of the digital economy influences the centralization of global value chain networks.

Subsequently, in the revised manuscript, we elucidate the mechanism through which the digital economy, after incorporating data elements into production factors, influences the centralization of global value chain networks. Once data elements are incorporated into the production function, they significantly boost enterprise productivity. From the perspective of the informatization mechanism, by leveraging the global internet and technologies such as big data, cloud computing, and the Internet of Things, enterprises can swiftly acquire extensive market information, customer demands, and supply chain data, enabling rapid data sharing and processing, thereby enhancing their total factor productivity. Furthermore, in terms of alleviating information asymmetry, the digital economy facilitates efficient information dissemination and sharing through internet platforms, making resource allocation more transparent. Consumers and enterprises can more conveniently access the information they need, reducing transaction costs and improving resource allocation efficiency. Ultimately, when enterprise productivity improves and resource misallocation is mitigated, their position within the global value chain will be elevated.

To provide a more intuitive illustration of the theoretical mechanism framework in this paper, we have further added a diagram of the theoretical mechanism framework in the revised manuscript. It is presented as follows:

Figure 1 illustrates the mechanism through which the development of the digital economy influences the centralization of global value chain networks. Specifically, the integration of data elements into firms' production activities enhances the centralization of global value chain networks by improving productivity and alleviating resource misallocation through these two channels.

Fig. 1 A Diagram Illustrating the Mechanism by Which the Development of the Digital Economy Influences the Centralization of Global Value Chain Network

**(2) Strengthen Causality Claims**

The study aims to address causality concerns, but there are still implied causal claims in certain sections (e.g., “The development of the digital economy significantly enhances a country's central location in the GVC network”).

While instrumental variables (IV) were used, discussing potential limitations of the IV approach would strengthen credibility.

**Response:** Thank you for your specific and professional comments and suggestions. This paper investigates the impact of digital economy development on the centralization of global value chain networks. As the review experts have pointed out, causal inference is one of the core elements of this study. Endogeneity, being the most critical issue that causal inference must confront, is addressed in this paper primarily through the instrumental variable method. Two main instrumental variables are employed: First, the number of landline telephones in 1984 for each country is utilized as an instrumental variable for the development of the digital economy; second, we constructed a weighted value combining the digital economy development level of other countries relative to the home country and the average digital economy development level of countries across different income brackets, multiplied by the digitization rate of various industries within each country. This serves as an instrumental variable for the digital economy at the "country-industry" level (for details, please refer to Table 4 and Table 5).

However, we acknowledge that despite addressing endogeneity issues through the instrumental variable method, there is still room for improvement in causal inference. This is an area where we need to make further efforts in the future.

**(3) Improve Flow and Readability**

Some sections, especially the methodology and theoretical model, are dense with mathematical formulations. Consider adding brief explanations or intuitive interpretations before presenting equations.

The literature review is comprehensive but could be structured better—perhaps by grouping studies into broader themes (e.g., digital economy impact on firms, trade networks, productivity).

**Response:** Thank you for your specific and professional comments and suggestions. Firstly, in terms of the readability of the article, we conducted a thorough review of the entire text, particularly focusing on the "methodology and theoretical model" sections, which contain a significant number of mathematical formulations. Therefore, before each formula, we provided a brief description of its meaning. For instance, before formula (1), we clarified that it represents a quasi-linear utility function; before formula (19), we explained that it is the baseline regression equation of this paper.

Subsequently, we reorganized the literature review. In accordance with the expert's suggestions, we restructured the literature review section, summarizing it based on the logical progression of the digital economy's impact on firms, trade networks, and productivity. The specific modifications are as follows:

The research related to this paper focuses on the following aspects: Existing research has explored the impact of the digital economy from multiple perspectives, most of which focus on the impact on labor force employment. First, the digital economy represented by AI has a strong substitution effect on the labor force particularly in traditional labor-intensive industries, inevitably leading to an increase in unemployment [9-11]and a decrease in the share of labor [12], but it also has a substitution effect on the traditional labor force in terms of creating new jobs [13]. Second, the digital economy has a significant boost to overall productivity, as it has strong technology-intensive attributes and a strong spillover effect compared to traditional industries, and hence it can have a significant boost to industry-wide productivity [14]. Finally, given the penetration of the digital economy, enterprises face digital transformation, and their production and operation activities are inevitably affected by the digital economy [15,16]. Most of the studies mentioned above have focused on the impact of the digital economy on the "binary margin" of firms' exports, but few studies have focused on its impact on the quality of export products [17]. Given the continuous penetration of the digital economy and the internet, Ma & Hu explored the impact of the digital economy on multiproduct exporters and found that information technology prices have been decreasing, reducing firms' export costs and improving the quality of their export products while also allowing them to spend more resources on producing their core products to maintain their competitiveness in the international market [18]. Additionally, research suggests that information and communication technology (ICT) plays a significant role in driving global value chain (GVC) activities[19]. Studies from both macro and micro perspectives have found that the development of the internet notably promotes GVC trade[20]. This promotion is mainly achieved by enhancing supply-demand matching and fostering communication and collaboration, injecting new vitality into the global value chain[21]. However, there are also opinions suggesting that ICT may have adverse effects on the upgrading of GVCs. Under the global value chain division system driven by labor arbitrage, the substitution effect of automation on labor leads to the contraction of GVC trade[22].

At the same time, there is a large body of literature using social network analysis as a research tool to analyze and measure the intricate trade issues among countries. The advantage of social network analysis is that it can reflect not only the overall trade pattern in the world but it can also reflect the importance and centralization of a country and each industry in the global trade network. If a country is at the center of the trade network, its access to resources and profit is stronger [2]. Traditional social network analysis usually considers each country as a point in the network, and the line linking the points is the bilateral trade flow. The analysis of the global trade network reveals that trade and economic exchange between countries are becoming more frequent [23,24]. Meanwhile, global trade networks are not static. Akerman & Sein studied the change in the global trade network in 1995-2011 and found that it is moving toward intensification, clustering, and decentralization [25]. In their analysis of the global trade network, Amighini & Gorgoni found that developing countries are playing an increasingly important role in global trade and that automated production is having an impact on previous trade patterns [26]. The above studies analyze the change in the global trade network mainly through bilateral trade volumes. However, with the changing division of labor systems in GVC, intermediate goods are often traded across multiple borders, so the value of a country's final export may originate from many other countries. Traditional social network analysis, which mainly uses total trade, has led to a misunderstanding of today's global trade pattern, so the GVC network should be reinterpreted using value-added trade [27]. To overcome the shortcomings of traditional trade statistics, value added flows are used as "edges" in trade networks, and thus, the construction of a global trade network is more in line with the characteristics of today's GVC trade [2,28].

The change in firms in terms of the division of production in GVC has been measured using the concept of the number of production stages[3]. Currently, the division of labor in GVC is facing many challenges, and COVID-19 has had a great impact on the global production supply chain [4-6], as it slowed the growth of the global economy and gradually reduced the incentive of countries to participate in the division of labor in GVC. The rise of artificial intelligence (AI) has had an impact on GVC, as the application of AI increased the efficiency of using intermediate goods to produce final goods [7], making the production process more automated and smarter, saving labor costs [8], and improving the division of labor of firms in GVC.

**(4) Refine Abstract and Introduction**

The abstract is clear but could be more concise in summarizing key findings.

The introduction could benefit from a stronger statement of research gaps and practical significance.

**Response:** Thank you for your specific and professional comments and suggestions. In the revised manuscript, we have made adjustments to the abstract, primarily by succinctly summarizing the research findings. We emphasize that the advancement of the digital economy elevates a country's centrality within the global value chain network, and this elevation is predominantly facilitated through the enhancement of productivity and the mitigation of resource misallocation. The specific revisions to the abstract are outlined as follows:

The development of the digital economy significantly relies on promoting economic liberalization and achieving high-end embedding of manufacturing into the global value chain (GVC). Based on the heterogeneous firm model, this paper introduces the factors of digital economy development, aggregates the production behavior of firms at the national level, and empirically examines the effects of digital economy development on the centralization of the GVC network and the internal mechanisms. The results show that the development of the digital economy significantly enhances a country's central location in the GVC network, primarily through enhancing productivity and alleviating resource misallocation. Meanwhile, digital economy development contributes more to the centralization of the GVC network in developed countries and high institutional quality industries. Further analysis shows that digital economy development not only enhances a country's central location in the GVC network but also extends the length of a country's GVC and promotes a country to climb up the GVC.

Additionally, in the Introduction section, we have supplemented explanations regarding the contributions of this paper to existing research, as well as its practical significance. The specific additions and modifications are as follows:

Despite the growing body of literature examining the impacts of the digital economy on economic growth, labor markets, and trade patterns, significant research gaps remain regarding its direct and indirect effects on the centralization of global value chain (GVC) network and the underlying mechanisms driving these changes. While existing studies have extensively explored how the digital economy facilitates the upgrading of GVC, mostly studies have largely overlooked its influence on the centralization of GVC network, which reflects a country's pivotal role and control over resources and information flows in the global economy.

In summary, this paper contributes to the existing literature by providing a nuanced understanding of the digital economy's role in shaping the centralization of GVC network. By integrating firm heterogeneity and examining both direct and indirect effects, we offer a comprehensive framework that elucidates the complex dynamics between digital economy development and GVC network centralization. Our research not only advances theoretical knowledge but also provides actionable insights for policy-makers aiming to leverage the digital economy for economic growth and global competitiveness.

**(5) Ensure Consistent Terminology**

Terms like "centralization of GVC networks" and "central location in the GVC" should be consistently defined and used throughout.

**Response:** Thank you for your specific and professional comments and suggestions. In the revised manuscript, we have standardized the use of professional terminology, particularly ensuring a consistent expression for "centralization of GVC network."

**Response to Reviewer7 :**

**(1)** In the introduction section, it can be further clarified to explicitly state the motivation for studying the impact of the digital economy on the centralization of global value chain networks. This should emphasize how, against the current global economic backdrop, the digital economy has emerged as a crucial factor in driving the enhancement of countries' positions within global value chains.

**Response:** Thank you for your specific and professional comments and suggestions. In the revised manuscript, we have provided supplementary explanations in the introduction, primarily adding the motivation behind the impact of the digital economy on the centralization of global value chain network. We have also emphasized the contributions of this paper to the existing literature and its practical significance. The additional content included in the introduction is as follows:

Despite the growing body of literature examining the impacts of the digital economy on economic growth, labor markets, and trade patterns, significant research gaps remain regarding its direct and indirect effects on the centralization of global value chain (GVC) networks and the underlying mechanisms driving these changes. While existing studies have extensively explored how the digital economy facilitates the upgrading of GVC, mostly studies have largely overlooked its influence on the centralization of GVC network, which reflects a country's pivotal role and control over resources and information flows in the global economy. In recent years, the digital economy, fueled by advancements in information and communication technologies (ICTs) such as the internet, big data analytics, cloud computing, and blockchain, has emerged as a transformative force reshaping the global economic landscape. This digital revolution is not merely confined to technological innovation; it is fundamentally altering the way businesses operate, goods and services are produced, and value is created and distributed across borders.

In summary, this paper contributes to the existing literature by providing a nuanced understanding of the digital economy's role in shaping the centralization of GVC network. By integrating firm heterogeneity and examining both direct and indirect effects, we offer a comprehensive framework that elucidates the complex dynamics between digital economy development and GVC network centralization. Our research not only advances theoretical knowledge but also provides actionable insights for policy-makers aiming to leverage the digital economy for economic growth and global competitiveness.

**(2)** In the data description section, the data in the text is up to 2014. It is suggested that the limitations of the data be mentioned in the discussion and that the updated data can be combined in the future to verify the sustainability of the conclusion.

**Response:** Thank you for your specific and professional comments and suggestions. The reason why the sample period of this study only extends up to 2014 is due to the limitations of the database used. The calculation of the core variables in this paper relies on the WIOD database, which, as of now, has only been updated up to 2014. Moreover, utilizing data from 2004 to 2014 for the issues examined in this paper can generally substantiate the conclusions of the research questions. Of course, once the WIOD database is updated in the future, we will also update the sample data for subsequent studies. In Section 4.3 "Data description and descriptive statistics" of the revised manuscript, we further highlight the limitations of the WIOD database and the potential for future improvements. The specific modifications are as follows:

(1) WIOD database

This paper uses the 2016 version of the WIOD database, including country-industry level input‒output data from 2004-2014, to calculate the core explanatory variables in the paper[35]. We also acknowledge that the current WIOD database is only updated up to 2014, which poses certain limitations to the research in this paper. If the WIOD database is updated in the future, further research can be conducted based on this paper.

**(3)** The robustness test section can be appropriately expanded to supplement and replace the robustness tests of samples (such as excluding countries or industries with extreme values).

**Response:** Thank you for your specific and professional comments and suggestions. In the revised manuscript, we have supplemented robustness tests, particularly focusing on the robustness checks conducted with respect to the sample. Finally, taking into account the impact of the 2008 financial crisis on the global economy, we re-ran the regression analysis using samples from after 2008. The results are presented in column (5) of Table 6, where the coefficient for the development of the digital economy is significantly positive at the 1% significance level, supporting the conclusions of the baseline regression.

**Table 6. Robustness test results.**

|  | **（1）** | **（2）** | **（3）** | **（4）** | **（5）** |
| --- | --- | --- | --- | --- | --- |
|  | **Substitution of explanatory variables** | **Substitution of explanatory variables** | **Consider trade liberalization** | **Remove outliers** | **Samples after 2008** |
| Digit | 0.0935^***^(0.0141) |  | 0.0877^***^(0.0325) | 0.0838^**^(0.0326) | 0.1998^***^ (0.0280) |
| Digit 1 |  | 0.0640^**^(0.0278) |  |  |  |
| Tarffic |  |  | 0.3241^**^(0.1263) |  |  |
| Country fixed effects | YES | YES | YES | YES | YES |
| Time fixed effects | YES | YES | YES | YES | YES |
| Industry - year fixed effects | YES | YES | YES | YES | YES |
| N | 8818 | 8818 | 8818 | 8713 | 5216 |
| R^2^ | 0.599 | 0.632 | 0.635 | 0.632 | 0.763 |

**(4)** The conclusions need to be more targeted. The current suggestions are relatively macro-level. It is recommended to refine the policy directions based on the research findings. For instance, for developing countries: propose strengthening digital infrastructure construction (such as 5G networks) and promoting labor skill training to address automation substitution; for industries with high institutional quality: suggest optimizing the intellectual property protection system to promote the deep integration of digital technology and manufacturing.

**Response:** Thank you for your specific and professional comments and suggestions. In the revised manuscript, we have made modifications to the policy recommendation section. Specifically, we have added policy suggestions for developing countries, emphasizing the need to strengthen digital infrastructure construction, optimize the intellectual property protection system, and promote the deep integration of digital technology with the manufacturing sector. The specific additions are as follows:

Finally, in response to the current situation of developing countries in the development of the digital economy and to enhance their position in the global value chain network, this paper recommends that emphasis should be placed on strengthening digital infrastructure construction. Measures such as expanding high-speed internet coverage and enhancing data center capabilities should be taken to provide solid support for the development of the digital economy. Simultaneously, it is crucial to optimize the intellectual property protection system, establish a legal and regulatory framework in line with international standards, strengthen law enforcement, and actively promote the deep integration of digital technologies with the manufacturing sector. This will stimulate innovation vitality and attract foreign investment. Through policy guidance and financial support, traditional manufacturing industries can be assisted in transforming and upgrading, thereby enabling developing countries to occupy a more advantageous position in the global value chain.

**Finally, we sincerely thank you again for reviewing this manuscript and providing us with valuable comments and suggestions for revising and improving the manuscript. We look forward to hearing from you in due time regarding this submission and to respond to any further questions and comments you may have.**

**Yours sincerely**

**The Authors**

**May 2025**
